# Slow modulations of high-frequency activity (40–140 Hz) discriminate preictal changes in human focal epilepsy

**DOI:** 10.1038/srep04545

**Published:** 2014-04-01

**Authors:** C. Alvarado-Rojas, M. Valderrama, A. Fouad-Ahmed, H. Feldwisch-Drentrup, M. Ihle, C. A. Teixeira, F. Sales, A. Schulze-Bonhage, C. Adam, A. Dourado, S. Charpier, V. Navarro, M. Le Van Quyen

**Affiliations:** 1 Centre de Recherche de l'Institut du Cerveau et de la Moelle épinière (CRICM) INSERM UMRS 975 - CNRS UMR 7225-UPMC, Hôpital de la Pitié-Salpêtrière, Paris, FRANCE; 2 Department of Biomedical Engineering, Universidad de Los Andes, Bogotá, COLOMBIA; 3 Bernstein Center Freiburg, University of Freiburg, Freiburg, GERMANY; 4 Freiburg Center for Data Analysis and Modeling, University of Freiburg, GERMANY; 5 Epilepsy Center, University Hospital of Freiburg, GERMANY; 6 Department of Neurobiology and Biophysics, Faculty of Biology, University of Freiburg, Freiburg, GERMANY; 7 CISUC-Centro de Informatica e Sistemas da Universidade de Coimbra, Faculty of Sciences and Technology, University of Coimbra, Coimbra, PORTUGAL; 8 Hospitais da Universidade de Coimbra, PORTUGAL; 9 AP-HP, Epilepsy Unit, Groupe Hospitalier Pitié-Salpêtrière, FRANCE

## Abstract

Recent evidence suggests that some seizures are preceded by preictal changes that start from minutes to hours before an ictal event. Nevertheless an adequate statistical evaluation in a large database of continuous multiday recordings is still missing. Here, we investigated the existence of preictal changes in long-term intracranial recordings from 53 patients with intractable partial epilepsy (in total 531 days and 558 clinical seizures). We describe a measure of brain excitability based on the slow modulation of high-frequency gamma activities (40–140 Hz) in ensembles of intracranial contacts. In prospective tests, we found that this index identified preictal changes at levels above chance in 13.2% of the patients (7/53), suggesting that results may be significant for the whole group (p < 0.05). These results provide a demonstration that preictal states can be detected prospectively from EEG data. They advance understanding of the network dynamics leading to seizure and may help develop novel seizure prediction algorithms.

In spite of available drug and surgical treatment options, a third of individuals with partial epilepsy have intractable seizures. The unpredictability of seizure occurrence limits their daily life, and underlies an enhanced risk of sudden unexpected death or morbidity^1^. Multiple quantitative analyses of the electroencephalogram (EEG) have aimed to define independent features and reproducible patterns that herald seizure onset^2^,^3^,^4^,^5^,^6^. Several studies have suggested that epileptic seizures do not occur randomly, but rather emerge from slow preictal changes in brain excitability that evolve over long timescales and predispose the brain to seizure^6^. In particular, a recent study - using intracranial electrodes connected to a telemetry unit implanted in ambulatory patients - showed that a prospective seizure prediction is possible with the knowledge of the patient's pre-seizure EEG patterns^7^. In most of the investigated patients, the warning device can successfully identify seizure occurrences in advance (from minutes to hours) and at a level better than would be expected on the basis of chance alone. However, despite encouraging results, this study has been limited by the small number of patients and complex machine learning algorithms making difficult to explore possible underlying physiological mechanisms.

Greater insights into the neuronal mechanisms of population behavior during the preictal period could not only improve seizure prediction but also help understand pathophysiological process of seizure initiation in the human brain^8^,^9^. Recent wide-bandwidth records of local field potentials made with intracranial electrodes during presurgical evaluation have identified several new classes of electrographic activity at high-frequencies. These low-amplitude activities occur at frequencies >40 Hz, are confined to millimeter-scale tissue volumes, and thus are less visible and often confused with eye movement or muscle artifacts in standard scalp electroencephalography (EEG)^10^,^11^. High-frequency activities, including gamma oscillations at 60–150 Hz and fast ripples at 250–500 Hz, have been associated with the initiation of epileptiform potentials and seizures in human temporal lobe and neocortical epilepsies^12^,^13^,^14^,^15^. Furthermore, while high-frequency oscillations are present intermittently throughout interictal period^16^,^17^,^18^, the occurrence of high-frequency activities increases significantly from several seconds to minutes before seizure onset^19^,^20^,^21^,^22^,^23^. Thus, high-frequency activities may help identify periods of increased predisposition to clinical seizures^4^. Nevertheless, recent investigations suggested that the relationship between high-frequency activities and seizure activity several minutes before the onset is variable, and no clear trend was observed^24^, especially in light of the many confounding variables such as fluctuating patient state^4^. Epileptic and physiological high-frequency oscillations are difficult to distinguish^25^,^26^. Both fluctuate strongly in amplitude over a time scale of several hours^17^ and the energy of high frequency activity changes with behavior with a maximal expression during seizure-free slow-wave sleep^27^,^28^,^29^.

The coupling between slow cortical potentials and high-frequency activities may provide novel insights into cortical network excitability^30^. Slow oscillations seem to be able to trigger and group local high-frequency oscillations. These cross-frequency couplings have been termed phase-amplitude coupling or “nested” oscillations and they have been suggested to represent a signature of cortical activation^31^,^32^,^33^,^34^,^35^. In this context, human intracranial EEG studies have identified a spatially distributed modulation of cortical high frequency oscillations in the gamma band (40–120 Hz) by theta oscillations (4–8 Hz)^36^,^37^ and slow waves (0.5–3 Hz)^29^. Cross-frequency coupling of high-frequency sub-bands in the gamma range to low-frequency electrical stimulation recorded with intracranial electrodes in patients with temporal lobe epilepsy has provided important clues on seizure susceptibility^38^,^39^. These observations led us to make a systematic study of preictal changes in cross-frequency coupling in intracranial EEG records from patients with medication-resistant partial epilepsy.

We assessed the sensitivity of cross-frequency coupling changes – defined as the ratio of correctly predicted seizures to the number of seizures investigated – and also their specificity from long-term interictal data^3^. Analyses were made from a large database of 53 patients with continuous multi-day intracranial EEG, collected in three European epilepsy centers (Freiburg, Germany; Paris, France; Coimbra, Portugal)^40^. Our analysis focused on interactions between the phase of low frequency rhythms (slow waves and theta) and the amplitude of different sub-bands of gamma rhythms. This analysis of spatial fluctuations in the coupling phase of ensembles of intracranial contacts revealed preictal changes, occurring at levels greater than chance in a small but significant number of patients. To validate our results, we compared them with predictions based on the power in individual frequency bands (delta, theta and gamma). We found that spectral powers did not lead to similar performances, suggesting that the coupling of different frequency bands is specific to the reported preictal changes.

## Results

**Figure 1:**
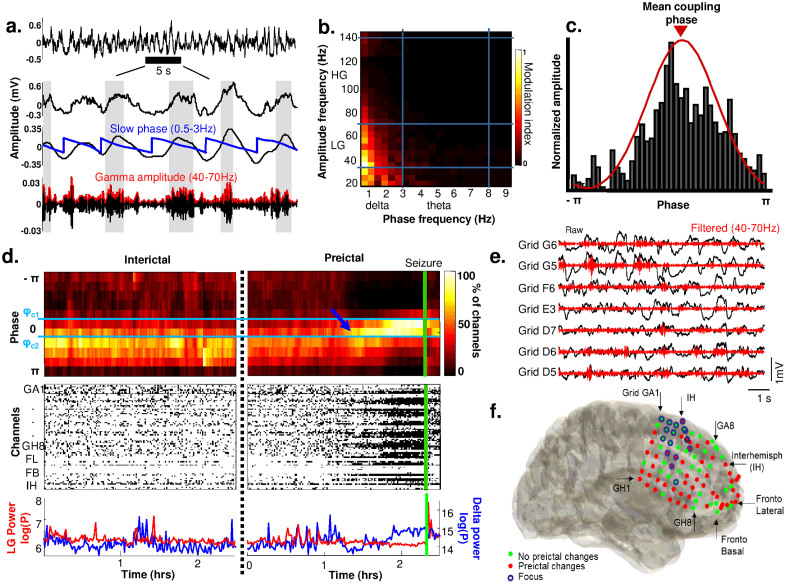
Preictal changes in phase relations of gamma and slow activity in an ensemble of intracranial signals. (a). Raw and filtered signals in the low frequency (0.5–3 Hz) and gamma frequency (40–70 Hz) bands with the respective instantaneous phase and envelope. (b). Phase-Amplitude modulation plot of a 20-minute preictal recording, showing the cross-coupling between different low and high frequencies. The colors depict the modulation strength as defined in Ref. 32. (c). Average amplitude of high frequency activity at different bins of low frequency phase. The mean of the distribution gives the *mean coupling phase*. (d). *Top*: For representative interictal and preictal segments, the proportion of channels with a coupling phase in the interval [*ϕ_c1_*, *ϕ_c2_*] is depicted in color. The vertical green lines indicate seizure onset. *Middle*: black points correspond to the contacts within this interval. *Bottom*: Power in delta and low gamma bands, shows preictal changes were not induced by an increase in gamma activities. (e). Preictal raw signals showing a strong cross-frequency modulation. (f). Spatial distribution of contacts (in red) implicated in the preictal changes projected on post implantation MRI scans. Implicated contacts were defined when incidences within the interval [*ϕ_c1_*, *ϕ_c2_*] were 2 times higher than the interictal average. Blue circles indicate contacts in the seizure onset zone.

Figure 1a shows a representative subdural electrocorticographic signal recorded 2 minutes before a seizure that occurred during slow-wave sleep (Id: 13, Table 1). The raw and filtered signals in the low gamma (LG) range show the recurrence of bursts of high-frequency activity occurring preferentially at the depth-positive phase of cortical slow waves. The strongest modulation for signals from this electrode was detected between the slow wave phase and LG amplitude (Fig. 1b), supporting previous findings that slow oscillations modulate gamma power in cortical recordings^28^,^29^,^30^,^31^. This cross-frequency coupling was quantified by constructing a histogram of LG amplitude over the phase of slow activity, showing a unimodal distribution^41^ (Fig. 1c). The single peak confirmed that gamma oscillations occurred preferentially at a mean coupling phase (*ϕ_c_*) close to 0 rad with respect to the intracranial slow waves. We tracked temporal changes in the coupling phase over ensembles of intracranial contacts by extracting *ϕ_c_* for each contact in consecutive non-overlapped 1-minute windows. Figure 1d illustrates a map of the temporal evolution of the number of contacts with a specific phase bin during an interictal state recorded 5 hours before a seizure. It shows that most contacts had a similar phase of coupling around 0.5 rad, which remained stable over several hours. However, tens of minutes before the seizure, large and consistent preictal deviations towards 0 rad were observed at multiple locations. Conventional power spectral analyses were less sensitive to these preictal changes (Fig. 1d). Visual inspection of the signals confirmed that high-frequency activities were strongly modulated, during the preictal state, by slow waves in many channels (Fig. 1e). Projected on post implantation MRI scans, contacts with these specific preictal phase fluctuations were distributed over spatially broad regions both within and outside the focal region (Fig. 1f).

**Figure 2:**
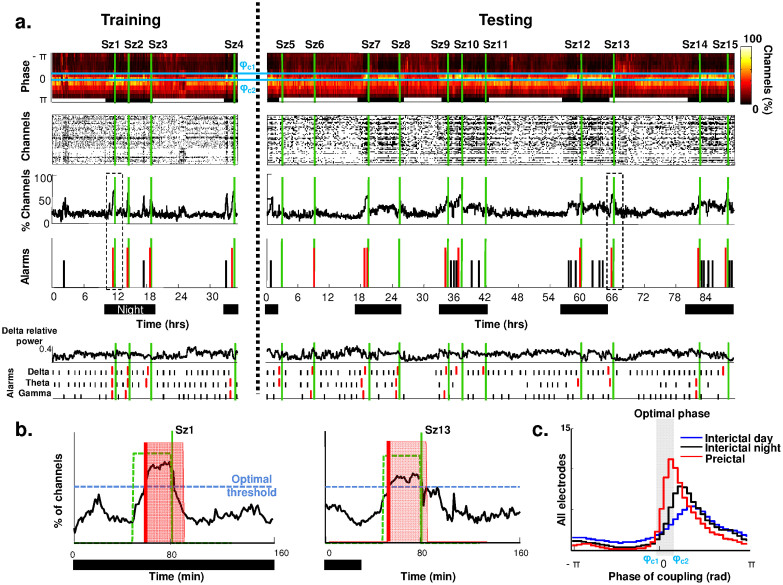
Prospective preictal state detection from dynamics of the mean coupling phase. (a). *From top to bottom*: Proportion of electrodes with a specific coupling phase over time (11 phase intervals; vertical green lines indicate seizure onsets); raster plot of contacts with a coupling phase in the interval [*ϕ_c1_*, *ϕ_c2_*] (*horizontal blue lines*); total proportion of channels over time; alarm triggered when the proportion of channels crosses a selected threshold. Several parameters of the algorithm were optimized from data of the training period, before the optimal selection was tested on new data from the testing period; Triggered alarms of the same prediction strategy but applied to the power in individual bands delta, theta and gamma. (b). Triggered alarms for two different seizures (*green lines*), followed by a refractory time equal to the assumed preictal duration. Vertical red lines show raised alarms and green dashed lines indicate the preictal interval. (c). Histogram of phase of coupling during preictal, interictal/day and interictal/night periods reflect the changes of coupling phase prior to seizures.

We investigated long-term coupling between fast and slow oscillations (see Methods for a detailed description of the strategy). First, the proportion of intracranial contacts at a specific mean phase interval was estimated over multi-day recordings. Second, an alarm was raised when this proportion exceeded a critical threshold. The threshold was adjusted and parameters including preictal durations and frequency bands were optimized on a first training part of the data obtained from each patient. For the selected optimal parameters, the last part of data was used to test prospectively discrimination between preictal from non-preictal periods. Performance results are reported for this test period, with parameters optimized from the training period. We tested the statistical significance of any changes, by comparing performance with a random predictor, which does not exploit any information contained in the EEG data^42^. Figure 2a–b shows a long-term analysis of data from the same patient as in Figure 1. Using a preictal duration of 60 minutes, optimal for the training period, prospective performance was above chance level. Our analysis gave values of sensitivity (SS) of 73% and false prediction rate (FPR) of 0.29/h, suggesting that most seizures were preceded by a specific preictal state and the false prediction rate was low. In this patient, seizures occurred predominantly during the night (10 pm–7 am), suggesting that sleep modulated seizure susceptibility. Nevertheless, preictal changes were present before daytime seizures (see sz13 in Fig. 2b) and such changes were not always evident before night seizures (see for example the testing phase between 18 and 24 hours in Fig. 2a). Moreover, the preictal phase distribution shifted to another phase configuration (from 0.5 to 0 rad), absent from seizure-free night periods, suggesting that this phenomenon did not reflect a simple sleep stage (Fig. 2c). To further confirm that our results did not mainly reflect power fluctuations related to different states of vigilance, we compared them with predictions based on the relative power in individual frequency bands (delta: 0.1–4 Hz, theta: 4–8 Hz, gamma: 30–140 Hz). Fig. 2a (Bottom) shows the alarms triggered by applying a threshold to the spectral powers averaged over all channels and following the same strategy as that using cross-frequency coupling. For this patient, power-based predictions were less sensitive and specific to preictal changes. In particular, it can be observed that delta activity was continuously dominant in the EEG and only slightly modulated by sleep (Fig. 2a).

Table 1 shows the parameters selected for the whole group of 53 patients, as well as the performance (SS and FPR) of the algorithm. Performance varied strongly between patients, presumably due to diverse etiologies, exogenous triggers, and the inherent physiological variability. Even so, compared to a random predictor, significant preictal changes were identified prospectively at above chance level in 13.2% of the patients (7/53). Based on a binomial approximation (see Methods for details), this result is significant for the whole group of patients at the 5% level (*P_binom_*{7,53,0.05} = 1.6% which is less than 13.2%). For the 7 patients with statistical significant results, that average sensitivity was 68% (range: 36–100%) and the FPR was 0.33/h (range: 0.08–0.72/h). There was a preference for slow wave/LG coupling in 5/7 patients where preictal phase identification was successful (Table 1). All significant cases corresponded to epilepsies with temporal or frontal foci. A third of cases (33%) were significant for patients with frontal lobe epilepsy, and only 10% were significant for cases of temporal lobe epilepsy. Significant performances during the test period, were comparable for patients with temporal lobe (SS = 66% and FPR = 0.33/h) and frontal lobe epilepsy (SS = 62% and FPR = 0.27/h). For all data sets from the different patients, we varied the duration of preictal window lengths on the training period and found an optimal duration of 60 minutes in 6/7 patients with significant performances. Finally, we found no substantial differences in prediction of seizures occurring during the night and during the day for the significant patients: 44% of seizures occurring during the day and 56% of night-time seizures were predicted). Similar performances were observed for nocturnal or day-time seizures (10 pm-7 am; SS = 73% and FPR = 0.25/h during the night; SS = 64% and FPR = 0.19/h during the day). To confirm the specificity of our results, we compared them with predictions based on the power in individual frequency bands. Only 4 patients had significant results for delta (SS = 64% and FPR = 0.15/h), 2 for theta (SS = 50% and FPR = 0.07/h) and 1 for gamma band (SS = 62% and FPR = 0.3/h). These results suggest that the performances of our detector are relatively independent from the different states of vigilance. In addition, the coupling between these different rhythms carries more information about preictal changes than the information of individual frequency bands taken separately.

**Figure 3:**
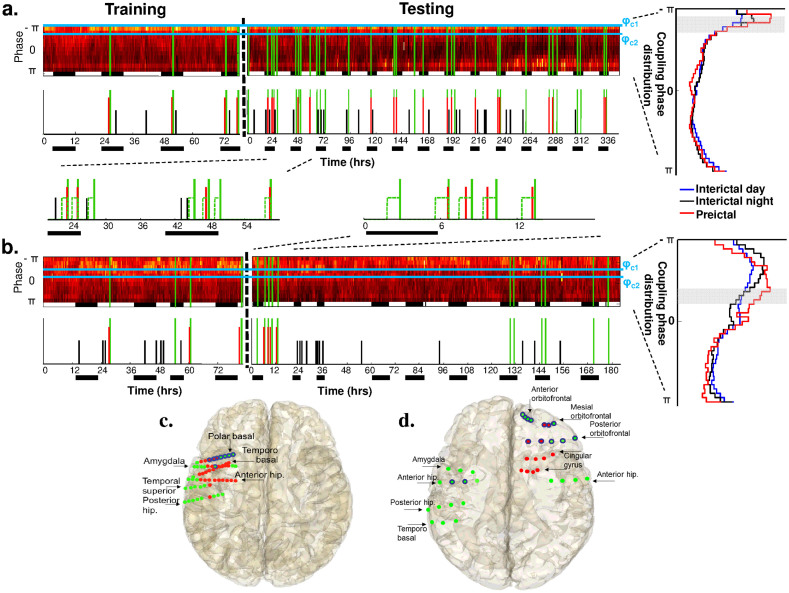
Data from two representative patients with statistically significant results. (a–b). Proportion of electrodes with a specific coupling phase over time (*top*) and alarms triggered when the proportion of channels crosses a selected threshold (*bottom*). Green lines depict seizures. Alarms linked to correct predictions shown in red and false positives in black reveal a high sensitivity and specificity. In (a) (*left*), the histogram of the coupling phase for 3 different periods (preictal, interictal/day and interictal/night) showed no significant phase variation but a preictal increase in the number of implicated contacts. In (b) *(left*), the histogram of phase of coupling shifts clearly during the preictal period. (c–d) Spatial representation of contacts for the two patients shown in A and B. Projected over the MRI reconstruction, contacts in the epileptic focus are indicated in blue. Contacts where the phase changed significantly to the optimal phase during preictal periods are indicated in red. Contacts outside the focus which did not change during the transition are depicted in green. Note that, despite a widespread spatial distribution, the contacts remained close to the focal regions.

Finally, we made a detailed analysis of the coupling phase distribution during preictal/interictal states in the 7 patients with statistical significant results. Two distinct types of phenomena emerged. In most cases (5/7), the preictal and interictal phase distributions were very similar except that wider brain regions were involved during preictal periods. Figure 3a shows a representative case (Id: 20 in Table 1). In this patient with mesial temporal epilepsy, seizures occurred preferentially at night, and analysis of slow wave/LG phase coupling revealed a low FPR (0.1/h) and a high sensitivity (56%). The preictal phase distribution was centered around −*π*, close to the one detected in day or night-time interictal states but with a larger involvement of intracranial contacts (Fig. 3a, right). In contrast, the preictal phase distribution shifted towards a different preferred phase in a minority of cases (2/7). Figure 3b shows a representative case (Id: 46 in Table 1). In this patient with frontal lobe epilepsy, the delta/HG phase coupling was selected to give the best performances with a remarkable high specificity (SS = 36% and FPR = 0.08/h). As for the patient of Fig. 1–2, pre- and interictal phase dynamics had distinct modulation properties and preictal shifts towards -0.9 rad were observed at multiple locations. In all significant cases, preictal changes occurred in regions close to but outside the seizure onset zone, indicating a widespread and diffuse spatial distribution (Fig. 3c, d).

## Discussion

Our study evaluated the feasibility of long-term seizure forecasting using intracranial EEG in a multicenter group of epilepsy patients with continuous long-term recordings (total analyzed duration of 531 days with total 558 clinical seizures). Bias introduced by selecting epochs for analysis was removed by using continuous multi-day EEG records from each patient^3^. Furthermore, as suggested^3^,^43^, we used a first part of available data for training and adjusting parameters for each patient and a second part to test prediction quasi-prospectively on unselected data. Finally, since our data was obtained with different EEG acquisition systems in three different hospitals, the evaluation provided a realistic sample of the heterogeneity of clinical circumstances and focal epileptic syndromes^44^. We found that a measure of brain excitability based on the coupling between low-frequency phase and high-frequency amplitude was able to identify preictal states for a significant number of patients (13.2%). Therefore, our observations demonstrated that a quasi-prospective analysis can distinguish between preictal and non-preictal states on long-term intracranial EEG recordings.

The existence of a preictal state in partial epilepsy is debated^6^. Despite several encouraging results, only very few studies have shown that a (quasi-) prospective prediction algorithm can perform above chance level^3^,^4^. For example, a forecasting algorithm using multiple spectral power bands as features and support-vector machine classification was tested on intracranial recordings of 18 patients (433 hours; 80 seizures) and reported a high sensitivity (97.5%) and low false prediction rate (0.27 per hour)^43^. In particular, the power changes in gamma bands (including the low and high gamma: 30–128 Hz) have been shown to be very relevant for prediction. Similarly, another study reported sensitivities of 0–100% and false prediction rates of 0–1.67/h in 6 patients based on the energy and entropy of high frequency activity (50–450 Hz)^45^. Nevertheless, the EEG recordings in these two studies were not continuous, with gaps between interictal and preictal segments, and data containing artifacts were sometimes removed. These procedures weaken long-term evaluation of the prediction performance during changing physiological or epileptic states. Therefore, although these studies may present higher sensitivity and/or specificity than ours, these results are not directly comparable. Recently, using a chronically implanted device running an algorithm which was trained on the basis of each patient's EEG signals, Cook et al.^7^ demonstrated that prospective seizure prediction is possible on long-term recordings of several months. The algorithms used in this work relied purely on classification of power measures in different frequency bands (8–128 Hz) and derived from subdurally recorded neural activity. During the prospective evaluation, the warning device worked to a level better than would be expected by chance in 8 over 11 patients. Using the same implanted device, but in dogs with naturally occurring epilepsy, a similar seizure forecasting algorithm performed significantly better than a random predictor in all investigated cases^46^. In our study, we used data from a large group of patients obtained during clinical monitoring for epilepsy surgery. Based on this database, we were only able to find a small group of patients having seizures that can be forecasted better than chance. Our observation seems to contrast with Cook's optimistic observations using long-term outpatient “real life” recordings and reporting high prediction performances in most of the investigated patients. One reason of this discrepancy could be the known limitations related to use of inpatient intracranial EEG data to evaluate prediction algorithms, including relatively short duration of EEG recordings (in particular for training purposes) and the effects of drug changes or surgery. Also, differences in statistical thresholds could explain different performances. Nevertheless, both studies support the feasibility of long-term seizure forecasting.

Statistically significant results were obtained in a small group of patients, which does not facilitate a full identification of pathophysiological mechanisms. Nevertheless, we resolved two types of preictal phenomena. In the first type (see data from patients of Fig. 2 or Fig. 3b), the preictal phase distribution shifted toward a distinct preferred phase. This situation may reflect the existence of a specific pathological state distinct from physiological states and independent of changes in the state of vigilance. Alterations in the modulation of high-frequency power may provide an indirect access to pathological spiking activities^47^ and reflect abnormal interactions between coupled networks^48^. In the second type of preictal phenomena (such as that shown in Fig. 3a), their phase distributions were very similar than those during interictal states but involved more distant brain regions. This may reflect a widespread increase in brain excitability, thereby a global state that is more susceptible to seizures. Clinical evidence suggests that certain normal states, particularly related to sleep and arousal, can favor seizure occurrence in epilepsy patients. For example, most nocturnal partial seizures occur during slow wave sleep while few ictal events occur during rapid eye movement (REM) sleep^49^,^50^. These preictal states may not reflect a deterministic, transitional state that inevitably leads to seizure, but a permissive “pro-ictal” state conforming to a normal brain state with a higher probability of seizure generation^51^. Thus, the high-level of false-positives detected by our algorithm may reflect underlying physiological mechanisms rather than short-comings of our detection that tend to label distinct events as similar. Nevertheless, as shown by our comparison with the power fluctuations in several frequency bands, there is not one to one correspondence between preictal changes and specific states of vigilance; this complex relationship is an area of ongoing research^52^.

We have shown that cross-frequency coupling can provide new insights into the transition from interictal to ictal states, but clinical applicability will depend on several other factors. False seizure prediction rates greater than about 0.15 FP/h are generally felt to be unacceptable for clinical application^53^,^54^. Our most favorable analyses provided an average sensitivity of 68% and a specificity of 0.33 FP/h, insufficient for clinical application. In only one of seven patients with significant performance, we observed both a remarkably high specificity and sensitivity (Fig. 3a). Nevertheless, we are encouraged by these results and believe that the discrimination framework we present here may be improved in the future towards a clinically acceptable performance if combined with other seizure prediction techniques^55^.

## Methods

### Database

Long-term intracranial EEG recordings from 53 epilepsy patients (26 males; age range, 3–63 years; mean age: 32 years) suffering from medically intractable partial epilepsy were analyzed. The data were recorded in three different epilepsy units: Epilepsy Center, University Medical Centre of Freiburg, Germany; Unité d'Épilepsie of the Pitié-Salpêtrière Hospital, Paris, France; Hospitais da Universidade de Coimbra, Portugal. Data acquisition and database were performed in accordance with the approved guidelines of the local ethic committees (Ethik-Kommission der Albert-Ludwigs-Universität Freiburg; Comité consultatif sur le traitement de l'information en matière de recherche dans le domaine de la santé, Pitié-Salpêtrière University Hospital; and Ethics Committee of the Coimbra University Hospital, respectively). Patients gave a written consent for a research use of these data. Funded by the European Union, this database was established to compile high-quality, long-term continuous EEG data, enriched with clinical metadata^40^ (http://www.epilepsiae.eu/). A standardized EEG annotation protocol was developed to ensure the comparability and reliability of seizure onset times at all project sites^56^. A total of 531 days of EEG records (~10 days/patient) included 558 seizures (~11 seizures/patient). Records include at least 5 days of continuous EEG from each patient with at least 5 clinically manifest epileptic seizures separated by more than 1.5 hours. EEG data were recorded using different digital video EEG systems with different sampling rates in the three different centres; Nicolet, 256 Hz; Micromed, 400 Hz; Compumedics, 512 Hz and Neurofile NT, 1024 Hz. Subclinical intracranial electrographic events were not analyzed. Table 1 shows patient characteristics, recordings details and locations of the seizure onset zone.

### Measuring the mean coupling phases

Cross-frequency coupling was defined by a statistical measure of interaction between different frequency bands. Specifically the phase of a slow oscillation was compared with the amplitude of a fast oscillation. The highest amplitude occurred at the so-called mean coupling phase. In accordance with previous human studies^29^,^36^,^37^, we investigated the coupling between delta (0.5–3 Hz) or theta (3–8 Hz) for low frequency phases, and low gamma (LG: 40–70 Hz) or high gamma (HG: 70–140 Hz or 70–120 Hz when sampling frequency was 256 Hz) for high frequency amplitude envelopes. We extracted coupling by implementing an algorithm previously proposed^41^, where raw signals were first filtered in the bands of interest, and then phase and amplitude were extracted using the Hilbert transform. Raw signals were filtered for frequency bands of interest using an 8-order Butterworth forward-backward IIR filter to avoid phase delays and improve frequency selectivity. Instantaneous phase *ϕ(t)* and instantaneous amplitude envelope *A(t)* were then extracted for delta (*ϕ_δ_*) and theta (*ϕ_θ_*), and for LG (*A_LG_*) and HG (*A_HG_*), respectively. The Hilbert Transform produces an analytical representation of the signal, with real part *x(t)* corresponding to the filtered signal and complex part y*(t)*, its phase-delayed version by π/2: 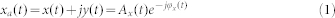
In this complex representation, the angle and magnitude of the transform correspond respectively to the instantaneous phase and envelope of the filtered signal (see Fig. 1a for an example). The modulation between four possible low phase – high amplitude pairs (*ϕ_x_(t) A_x_(t)*), was computed as follows. First, the range of the phase signal *ϕ_x_(t)*, with values within the interval *(−π,π)*, was divided into bins *ϕ_i_*with *i = 1,…,40*. Then, the time indexes *k_i_*when the phase fell in the interval *ϕ_i_ ≤ ϕ_x_(k_i_) < ϕ_i+1_*, were determined. For each bin, amplitudes at time *k_i_* of the high frequency time series were averaged (*<A_x_(k_i_)>*). The distribution of mean amplitudes versus phase defined the phase of the slower activity, where high frequencies tend to appear (Fig. 1b). We quantified this tendency by approximating the distribution to a Von Mises function (circular Gaussian), and extracting the mean phase representing the preferred phase of coupling *ϕ_c_*. A statistical threshold (based on a phase randomization procedure for instance) was not used^41^. We thus maintained computationally efficiency in treating data from data epochs of 1 minute. Codes are freely available in the EPILAB platform^55^ at: http://www.epilepsiae.eu/project_outputs/epilab_software/.

### Identifying fluctuations of mean coupling phase patterns

We followed the time course of mean coupling phases of multiple contacts, during consecutive non-overlapping time windows of 1 minute, by determining the proportion of contacts with a mean coupling phase at a specific phase interval [*ϕ_c1_,* *ϕ_c2_*]. Figure 1c shows an analysis for a representative patient during interictal and preictal periods. Long-term trends of coupling phase were determined by applying a first-order Kalman filter to the proportion of electrodes within [*ϕ_c1_,* *ϕ_c2_*]. Thus short-time fluctuations were smoothed, as previously proposed^43^. A variable threshold was then set to produce an alarm when the number of intracranial contacts exceeded a defined value (Fig. 2a, b). After an alarm, a new alarm could be generated only after a refractory period corresponding to the defined preictal time.

### Prospective evaluation of preictal changes

Application of highly optimized methods to small and preselected data may result in overestimating the results that are poorly reproducible on unselected, longer continuous data^3^. While algorithm parameters may be adjusted to data from an individual patient, this optimization must be performed only on one part of the data (*the training set*) excluded from the data set used subsequently to assess algorithm performance (*the testing set*). For training, we used continuous data including the first 4 seizures and at least 10 hours of recording. This training data permitted parameter selection to optimize sensitivity and specificity. We determined optimal values for the coupling band (among 4 pairs: slow waves or theta versus LG and HG), phase interval [*ϕ_c1_,* *ϕ_c2_*] (among 11 intervals) and preictal duration (10, 30, 60 min). Statistical performance was calculated, as usual, in terms of sensitivity (SS, ratio between correct detections and total seizures), and false prediction rate (FPR, number of false predictions per hour). Optimization was performed such that the performance was the closest to the optimal value (SS of 100%, FPR of 0), with distance measured by 
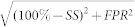
. Finally, the test set containing unknown data and seizures was used to derive a prospective statistical assessment of performance of the optimized algorithm. FPR was defined as the number of false alarms during the test set divided by the duration in which false alarms could be triggered (with refractory periods excluded), which is obtained by subtracting the time under false warning from the total interictal period^3^. Mathematically, the FPR is expressed as: 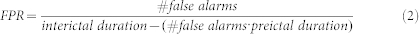


### Comparison with a random predictor

Once the sensitivity and specificity of a prospective prediction algorithm has been assessed on the test dataset, it is necessary to ask whether it is indeed superior to the chance level. We evaluated statistical significance by comparing results to chance established by a random predictor, which produced alarms in the absence of information from the EEG signal^42^. With random alarm generation, the probability to predict at least *k* out of *K* seizures was given by a binomial distribution: 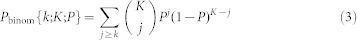
Where *P* indicated the probability (Poisson process) of that a single alarm was triggered during the preictal time. A unique predictor was used for the final evaluation of the testing phase of the prospective approach. Therefore the critical level of sensibility for the random predictor *σ_rand_* was stated as follows: 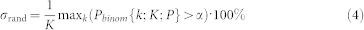
Where *α* represented the significance level of the predictor (*α* = 0.01 in this case). For the same value of FPR, the sensitivity of our method must be above *σ_rand_* to be superior to the random predictor, and thus reach statistical significance.

### Statistical evaluation on the whole group of patient

In order to test whether the observed results can be considered significant for a whole group of patients, the number of patients with statistical significant results can be calculated for the null hypothesis of no true predictive performance. For a significance level of *α*, the probability to observe, for at least *n* of *N* patients, sensitivities larger than *σ_rand_* follows a binomial distribution: 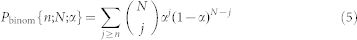


### Comparison to prediction based on the power of delta, theta or gamma bands

To evaluate whether our observations based on cross-frequency coupling did not simply reflect power fluctuations related to different states of vigilance, a similar strategy was developed to generate alarms based on the relative power of different bands: delta (0.1–4 Hz), theta (4–8 Hz) and gamma (30–140 Hz). After filtering the raw signals, the power spectral density was computed by Burg method over consecutive windows of 5 seconds. Then, the power of each band relative to the power in the whole frequency range was extracted. The averaged relative power over all channels was used to determine an optimal threshold above which an alarm of preictal period was triggered. As in our cross-frequency coupling strategy, the predictor based on the individual bands was first optimized on the training data (in sample definition of preictal times and thresholds). Finally, the algorithm performance (sensitivity and FPR) and the comparison with a random prediction were assessed on independent data (out-of-sample).

## Author Contributions

M.L.V.Q. designed the experiment; C.A.R., V.N. and M.L.V.Q. performed research; C.A.R., M.V., A.F. and H.F.D. analyzed data; C.A.R. and M.L.V.Q. wrote the manuscript; M.I., C.A.T., F.S., A.S.B., C.A., A.D. and S.C. contributed data/analytic tools. All authors reviewed the manuscript.

## Figures and Tables

**Table 1:** Patient information and results: the sampling frequency, focus, and number of channels is given for each patient. Focus is indicating by lobe (frontal (f), temporal (t), occipital (o), central (c), parietal (p)), by region (mesial (m), lateral (l), basal (b), posterior (p)), and by lateralization (right (r), left (l), both (b)). Optimal parameters obtained in training phase, as duration, number of seizures, preictal period length, coupling frequency band (L for low gamma, H for high gamma, D for delta and T for theta), threshold, and performances (SS and FPR) are indicated. Additionally, the duration, number of seizures and performances obtained during testing phase (SS, FPR and significant patients) are given. Last column shows the significant patients obtained when the same prediction strategy was applied to the power in individual bands delta (D), theta (T), or gamma (G)

				Training								Testing					
ID	Fs (Hz)	Focus (lobe/region/lateralization)	# Chan	Dur (h)	#Sz	SOP (min)	Frequency Band	Preictal phase (rad)	Thr %	SS %	FP/h	Dur (h)	#Sz	SS %	FP/h	Sig.	D/T/G
1	1024	f-r	98	126	4	60	LT	−2,62	30	75	0,38	120	6	100	2,04		
2	1024	t-l	96	135	4	60	HT	1,38	21	100	0,60	82	4	50	2,21		
3	1024	tml,t-r	57	64	4	60	LD	0,81	30	75	0,44	55	9	11	0,18		
4	1024	tmr,tlr	117	108	4	60	HD	0,24	21	75	0,36	75	5	20	0,37		
5	1024	tlr	38	171	3	60	HT	0,81	11	67	0,14	80	2	50	0,64		
6	1024	tml	74	99	4	60	LD	−0,90	19	100	0,45	63	5	100	0,72	*	
7	1024	t-l	46	85	4	60	HD	−2,62	11	75	0,59	163	5	80	4,35		
8	1024	t-l,f-l	115	42	4	60	LT	−2,05	21	75	0,42	86	8	63	0,44		
9	1024	tmr	62	16	4	60	HD	1,95	19	75	0,11	139	10	20	0,46		
10	1024	fbr,tm-	124	104	4	60	LT	−2,05	15	75	0,22	48	3	33	6,02		
11	1024	f-r	48	25	4	60	LT	−2,05	11	75	0,11	116	8	13	0,35		-**
12	256	tll	40	70	4	60	LD	1,95	22	100	0,10	129	13	54	1,11		
13	1024	f-r	93	35	4	30	LD	0,24	44	100	0,10	89	11	73	0,30	*	
14	1024	f-l	89	123	4	60	HD	2,52	30	100	0,30	122	12	75	0,77		*--
15	1024	o-r,t-l	114	29	4	60	LT	2,52	18	100	0,27	85	6	17	0,30		
16	1024	tlr,tmr	69	18	4	60	HT	1,95	36	75	0,09	153	11	73	0,43	*	
17	1024	tml,t-l	83	34	4	60	HT	−2,05	27	75	0,08	109	18	22	0,13		
18	1024	tmr,tlr	46	23	3	60	HD	2,52	42	100	0,06	201	10	40	0,33		
19	1024	f-l,c-l	83	25	4	60	HT	−2,05	7	75	0,24	85	13	31	1,72		
20	1024	tml	84	79	4	60	LD	−2,62	50	100	0,06	346	32	56	0,10	*	*--
21	256	tmr	54	61	4	60	HT	−2,05	19	100	0,30	164	18	6	0,31		
22	256	tbl,tll,--b	41	96	4	60	LT	2,86	39	75	0,36	71	2	0	0,33		
23	512	tml,t--	39	143	4	60	LD	1,38	25	75	0,52	21	2	50	0,71		
24	256	t-b	28	42	3	60	HT	2,86	47	67	0,22	92	2	0	1,37		
25	256	tml,tll,t-r	42	156	4	60	LT	2,52	39	100	0,68	41	3	67	1,31		
26	256	t--,f-l	36	72	4	60	HD	−1,48	25	75	0,46	108	16	63	2,35		
27	256	fml	47	180	4	60	LD	0,81	36	100	0,41	49	4	100	0,63	*	
28	256	tbr	96	74	4	60	HD	2,52	10	75	0,19	125	13	62	1,59		
29	256	tml,tll	84	87	4	60	HT	1,95	47	75	0,14	136	6	0	0,02		
30	400	tpr	52	390	4	60	HT	−1,48	27	100	0,41	74	3	67	0,27		
31	400	f-l	58	27	4	30	LT	−2,05	24	75	0,13	168	21	10	0,17		
32	400	fpl	38	210	3	60	HT	−0,90	36	100	0,44	136	2	100	1,17		
33	400	tml	42	117	3	60	HD	−2,05	22	100	0,33	120	2	100	2,22		
34	400	tmr	49	197	3	60	LT	−2,05	22	100	0,10	154	2	50	0,14		
35	400	t-r	51	124	3	60	HD	1,38	22	100	0,52	213	2	100	1,69		
36	400	tml	56	145	3	60	LD	−2,05	22	67	0,07	14	2	100	2,70		**-
37	400	tpr	48	177	4	60	HD	0,81	16	75	0,12	168	5	60	0,56		
38	400	o--	42	407	2	60	LT	1,38	22	100	0,20	37	2	0	0,03		
39	400	tml	32	155	4	30	HD	−1,48	22	75	0,25	69	3	0	0,31		
40	400	tlr	41	109	4	60	LD	0,81	19	75	0,39	39	4	50	2,31		
41	400	t-r,o-r	34	198	4	60	LD	−0,33	28	75	0,04	145	4	0	0,13		
42	400	f-r	28	62	3	60	LD	1,38	23	67	0,09	152	8	38	0,08	*	
43	400	p-r	33	170	4	60	LT	0,24	31	75	0,12	15	2	50	1,61		
44	400	tbr	32	508	2	30	HD	2,52	47	100	0,09	51	2	0	0,35		
45	1024	fbr,tmr	41	17	4	60	LD	−2,62	36	75	0,35	301	11	27	0,10		
46	1024	fbr,tml	29	82	4	60	HD	−0,90	34	75	0,12	185	11	36	0,08	*	
47	1024	h-l,fpl	63	131	3	60	HD	0,24	30	100	0,09	268	2	0	0,03		
48	1024	tmr	63	180	4	60	LD	1,95	24	100	0,41	265	4	100	1,79		
49	1024	tlr	60	403	2	60	LD	0,24	24	100	0,19	53	2	50	0,47		
50	1024	tm-	56	196	4	60	LT	2,52	24	75	0,16	291	9	0	0,15		
51	512	t-l	25	31	3	60	LD	−0,33	28	67	0,28	104	2	100	0,66		
52	1024	p-l	39	42	2	60	HD	0,24	42	100	0,29	42	2	50	0,48		
53	512	f-r	10	149	3	60	LD	−2,62	31	100	0,15	42	2	50	0,80		*--
